# Circulating tumor DNA dynamics using patient-customized assays are associated with outcome in neoadjuvantly treated breast cancer

**DOI:** 10.1101/mcs.a003772

**Published:** 2019-04

**Authors:** Timothy M. Butler, Christopher T. Boniface, Katie Johnson-Camacho, Shaadi Tabatabaei, Daira Melendez, Taylor Kelley, Joe Gray, Christopher L. Corless, Paul T. Spellman

**Affiliations:** 1 Department of Molecular and Medical Genetics, Oregon Health and Science University (OHSU) Portland, Oregon 97201, USA;; 2 Wellcome Trust Sanger Institute, Cancer Ageing and Somatic Mutation, Hinxton, Cambridgeshire CB10 1SA, United Kingdom;; 3 Department of Biomedical Engineering, OHSU Portland, Oregon 97201, USA;; 4 Knight Cancer Institute, OHSU Portland, Oregon 97201, USA;; 5 Center for Spatial Systems Biomedicine, OHSU Portland, Oregon 97201, USA;; 6 Division of Hematology and Medical Oncology, Portland Veterans Affairs Health Care System, Knight Cancer Institute, Oregon Health and Science University, Portland, Oregon 97201, USA

**Keywords:** neoplasm of the breast

## Abstract

Pathological complete response (pCR) is an accurate predictor of good outcome following neoadjuvant chemotherapy (NAC) for locally advanced breast cancer. The presence of circulating-tumor DNA (ctDNA) has recently been reported to be strongly predictive of poor outcome in similar patient groups. We monitored ctDNA levels from 10 women undergoing NAC for locally advanced breast cancer using a patient-specific, hybrid-capture sequencing technique sensitive to the level of one altered allele in 10,000. Plasma was collected prior to the start of NAC, prior to each infusion of NAC, and during follow-up for between 350 and 1150 d after the start of NAC. Prior to the start of NAC, ctDNA was detectable in 3/3 triple negative, 3/3 HER2^+^, and 2/4 HER2^−^, ER^+^ breast cancer patients. Total cell-free DNA levels were considerably higher when patients were on NAC than at other times. ctDNA dynamics during NAC showed that patients with pCR experienced rapid declines in ctDNA levels, whereas patients without pCR typically showed evidence of residual ctDNA after initiation of treatment. Intriguingly, two of three patients that showed marked increases in ctDNA while on NAC experienced rapid recurrences (<2 yr following start of NAC). The third patient that had increases in ctDNA levels while on NAC had low-grade ER^+^ disease and showed residual ctDNA after surgery, which became undetectable after local radiation. Taken together, these results demonstrate the ability of our approach to sensitively serially monitor ctDNA during NAC, and identifies a need to further investigate the possibility of stratifying patients who need additional treatment or identify therapies that are ineffective.

## INTRODUCTION

Approximately one in eight women will receive a breast cancer diagnosis in their lifetime (Howlader et al. 2014). Breast cancer can be divided into three major subtypes defined by the overexpression of estrogen receptor (ER^+^) and human epidermal growth factor 2-neu (HER2^+^) or their absence (triple-negative breast cancer [TNBC]). These subtypes also correlate with gene expression signatures and prognosis, with ER^+^ having a good prognosis and HER2^+^ and TNBC having a worse prognosis (Dai et al. 2015; Prat et al. 2015). Breast cancer is typically treated with chemotherapy combined with surgery and, if appropriate, an agent targeting estrogen receptor or HER2. This regimen results in 70% of patients remaining disease-free at 5 yr across all subtypes (EBCTCG 2012).

Neoadjuvant chemotherapy (NAC) has become an increasingly common treatment in breast cancer (Haddad and Goetz 2015). The first study testing this treatment approach showed that although patient outcomes were nearly identical, patients given NAC were more likely to receive a less aggressive, breast-conservation surgery. They were also less likely to have evidence of disease in the axillary lymph nodes (Rastogi et al. 2008). In addition, patients undergoing neoadjuvant treatment could be assessed for pathological complete response (pCR), the complete absence of disease following neoadjuvant treatment. Patients who achieve pCR have significantly increased disease-free and overall survival (Esserman et al. 2012; von Minckwitz et al. 2012). pCR rates are not uniform across subtypes, being both more common and having greater prognostic value in HER2^+^ and TNBC disease (Esserman et al. 2012; von Minckwitz et al. 2012; Prat et al. 2015). NAC is typically divided into two separate treatments given sequentially: The first treatment, referred to as AC, uses the DNA intercalator, doxorubicin, and the DNA cross-linker, cyclophosphamide; the second treatment uses the microtubule inhibitor, paclitaxel. These drugs are typically administered once every 2 wk for 3–6 mo. Paclitaxel is typically a better tolerated therapy than AC, so in drug trials adding new agents to NAC, the investigative drug is typically combined with paclitaxel and done prior to the AC arm (Park et al. 2016; Rugo et al. 2016).

Most patients receiving NAC have some response during the course of their treatment; however, a small subset shows no response (6%) or progression (3%) (Caudle et al. 2010). These patients could possibly benefit from stopping treatment and moving straight to surgery or switching to a different neoadjuvant treatment. Serial measurement of circulating-tumor DNA (ctDNA) is a method that has potential for on-treatment tumor monitoring and offers a promising tool in making such determinations.

ctDNA, a subfraction of cell-free DNA, is fragmented genomic DNA present in the blood plasma that is the result of apoptosis and necrosis of tumor cells (Crowley et al. 2013; Butler et al. 2017). Tumor-specific mutations can be reliably detected in the plasma of patients with cancer, and there is a positive correlation between disease burden and changes in ctDNA levels. However, studies to date have not evaluated this relationship in the neoadjuvant setting (Dawson et al. 2013; Murtaza et al. 2013; Madic et al. 2015; Tie et al. 2015). A potential use case for ctDNA detection and analysis is near-real-time monitoring of a patient's response to treatment. This would allow for rapid feedback as to whether a given therapy is working, thereby allowing for ineffective therapies to be adjusted or stopped. In addition, tracking multiple mutations of interest present in ctDNA may provide insight into whether certain mutations are being selected for or against during treatment (Murtaza et al. 2015; Abbosh et al. 2017).

In this study, we set out to measure ctDNA abundance before, during, and after neoadjuvant treatment to determine whether ctDNA levels and composition can predict response to treatment. We found that there is a dramatic reduction in ctDNA abundance during treatment in patients that is independent of pCR. In the three patients whose tumor size increased following treatment, we observed an increase in midtreatment ctDNA, suggesting that this approach may have promise as an early predictor of disease progression.

## RESULTS

### Study Design

**Figure 1.**
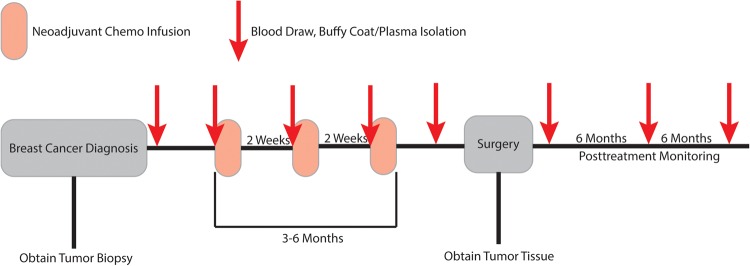
Plasma and tissue sampling strategy. Overview of study design collecting tumor tissue and plasma samples before, during, and after NAC. On-treatment plasma samples were taken in the infusion clinic before administration of drug.

**Figure 2.**
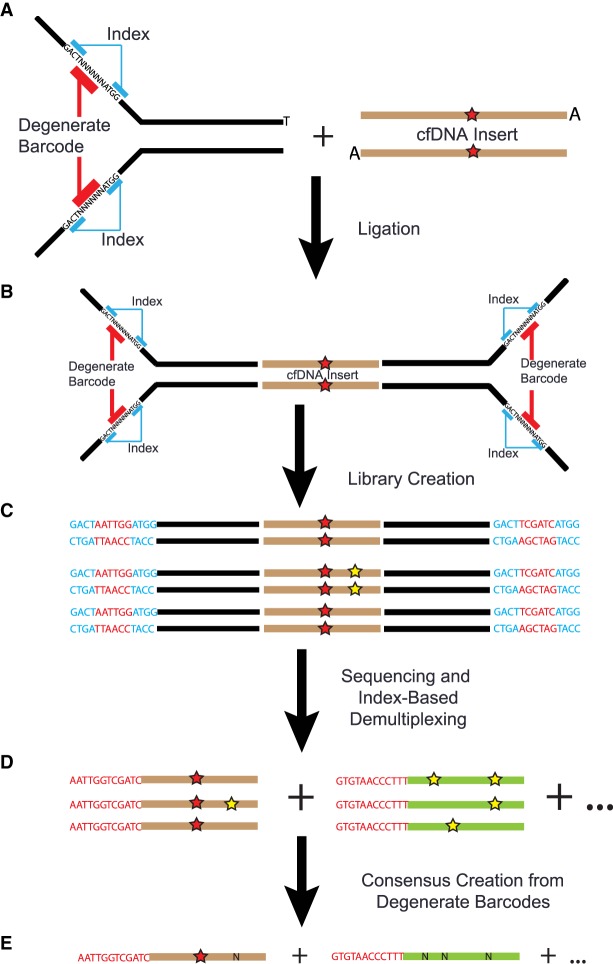
Overview of DIDA adapters, library creation, and consensus creation. (*A*) Schematic of T-tailed DIDA adapter; regions in black are standard Illumina adapter sequences. (*B*) Following ligation to A-tailed cell-free DNA (shown with a point mutation, red star) adapters are ligated to each side containing sample-specific index sequence and degenerate barcode. (*C*) Following library creation and amplification, multiple copies of the same template molecule are created; polymerase errors are shown with a yellow star. Blue sequences are known multiplexing indices, and red are the degenerate barcodes. (*D*) Following sequencing and demultiplexing, reads are grouped by mapping position and degenerate sequence. Brown and green represent copies of two different input molecules. (*E*) Reads are collapsed into a single-strand consensus sequence (SSCS), using mapping position and degenerate sequence; real mutations are retained, whereas polymerase errors are replaced by Ns.

In this study, ctDNA was quantified from time points taken before, during, and after NAC (Fig. 1). Breast cancer patients slated for NAC were identified and consented as part of two IRB approved studies, allowing for the collection of tumor tissue, serial blood draws, and access to medical charts for identification of clinical correlates. Ten patients were analyzed here in detail representing all three major breast cancer subtypes with three patients having achieved pCR (Table 1). In general, a 30–40 mL blood draw was collected before the start of NAC, prior to the start of each infusion appointment, prior to surgery, and at ∼6-mo intervals following surgery. As this was not a clinical end point, we were unable to obtain all plasma time points for all patients. Tumor tissue from the diagnostic biopsy was also collected and used for whole-exome sequencing to identify tumor-specific variants; 10 to 30 of these variant sites were used to generate target DNA sequences for a patient-specific hybrid capture panel used to quantify ctDNA levels in blood plasma. The serially collected plasma samples were sequenced using Dual-Indexed Degenerate Adapter (DIDA) unique molecular identifier sequencing (Fig. 2). A subset of the samples was also analyzed using Safe-SeqS, assaying each mutation individually (Kinde et al. 2011). The mutant allele frequencies in the ctDNA were compared against the patient's clinical data to determine if there was a correlation with treatment outcome.

**Table 1.** Enrolled patient characteristics

Patient	Age	Receptor status	Primary TNM	PathCR	Pretreat tumor volume (cm^3^)	Imaging post-treat tumor volume (cm^3^)	Path posttreat tumor volume (cm^3^)	Lymph node status	NAC treat-1	NAC treat-2	ctDNA pattern
BCR495	57	HER2^+^	cT1bN0M0	Yes	13.8	0	0	NA	TDM1 + pertuzumab	AC	Only detectable pretreatment
BCR494	43	TNBC	cT3N0M0	Yes	16.6	0	0	NA	Paclitaxel + ganitumab	AC	Undetectable midtreatment
BCR503	66	ER^+^PR^+^ HER2^+^	cT3N0M0	No	14	0	0	3/14	TDM1 + pertuzumab	AC	Undetectable midtreatment
BCR492	68	ER^+^PR^+^ HER2^+^	ypT2N2M0 (cT4N1 M0)	No	172	80	65	6/8	Paclitaxel + trastuzumab	AC	Undetectable midtreatment
BCR486	39	ER^+^PR^+^	ypT2N2M0 (cT2N0M0)	No	9	6.5	23	NA	AC	Paclitaxel	Increasing on-treatment
TB39	61	TNBC	ypT2N0M0 (cT2N0M0)	No	11	21.8	9 largest 2 dims	NA	Ganetespib + paclitaxel	AC	Increasing on-treatment
TB52	53	TNBC	ypT2N2M0 (cT3N0M0)	No	19	25	31	4/17	AC	(Carbo/paclitaxel given in adjuvant setting)	Increasing on-treatment
BCR488	54	ER^+^PR^+^	ypT1bNOMO (cT2NOMO)	No	20	0.8	50	NA	Trebananib + paclitaxel	AC	Only detectable pretreatment
BCR480	51	ER^+^PR^+^	ypT2bN0M0 (cT2N0M0)	No	16	6.3	1.9	NA	AC + paclitaxel	AC	Undetectable
BCR481	64	ER^+^	ypTONOMO (cT1 NOMO)	Yes	2	0	0	NA	AC	Paclitaxel	Undetectable

Pretreatment and imaging posttreatment tumor volume based on MRI measurement. Lymph node status indicates how many lymph nodes were positive from the surgical specimen.

(yp) Post-NAC pathology staging, (c) pre-NAC imaging staging, (TDM1) anti-HER2 treatment.

### Evaluation of Panel Performance

**Figure 3.**
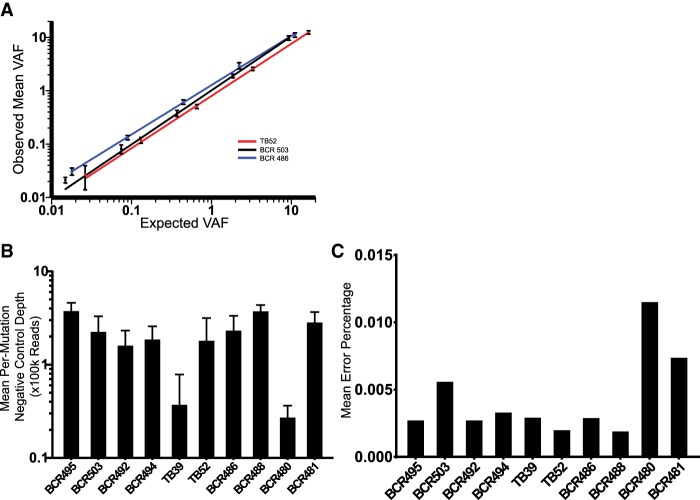
Hybrid capture panel performance. (*A*) Performance of three patient-specific mutation panels following serial dilution and error correction. Error bars ± SEM. (*B*) Mean per-mutation negative control depth for each patient-specific hybrid-capture panel. Error bars ± SEM. (*C*) Mean negative control error percentage for each patient-specific panel.

To determine the sensitivity and reproducibility of the patient-specific hybrid-capture panels, primary tumor DNA samples from patients TB52 (TNBC), BCR486 (ER^+^), and BCR503 (HER2^+^) were serially diluted into unmatched buffy coat DNA; the same buffy coat DNA was used for all three dilutions. These dilutions were conducted in triplicate, sequenced, and, following error correction, variant allele fractions (VAFs) were averaged across all mutations on the panel (Fig. 3). All three patient-specific panels performed well, with high degrees of reproducibility and a linear response down to low detection levels (Fig. 3A). We generated an average of 200,000× coverage (range 25,000–375,000) across our panels using a set of negative control samples with multiple library preparations sequenced multiple times (Fig. 3B). This allowed us to determine the panel-wide error rate for each patient and to identify those variants that had error rates of less than one input molecule in 10,000 (Fig. 3C). Because of the high accuracy of our error-correction technique around these specific mutations, they were used for subsequent ctDNA quantification and could therefore reliably detect tumor-derived ctDNA at a VAF at or <0.01% (Supplemental Table 1).

### Pretreatment ctDNA Concentration

**Figure 4.**
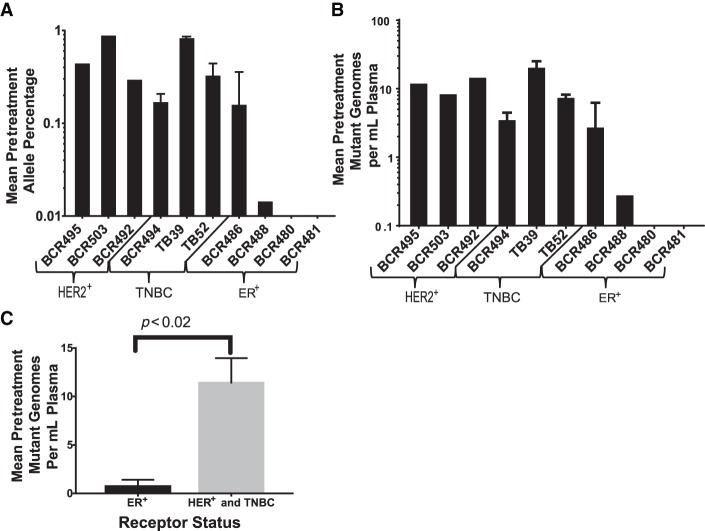
Pretreatment ctDNA levels. (*A*) Mean pretreatment ctDNA mutant allele percentage for 10 enrolled patients. Error bars ± SEM. (*B*) Mean pretreatment mutant genomes per mL of plasma for 10 enrolled patients. Error bars ± SEM. (*C*) Mutant genomes per mL of plasma grouped by receptor status ER^+^ HER^–^ (4) versus all other statuses (ER^+^ HER2^+^ [2], ER^–^ HER2^+^ [1], TNBC [3]). Error bars ± SEM, unpaired Student's *t*-test.

Using the patient-specific custom capture panels and our DIDA error-correction sequencing method, ctDNA was quantified in each pretreatment plasma sample (Fig. 4). ctDNA was detectable in eight out of 10 patients at mean allele fraction ranging from 0.01% to 0.91%. Our 80% detection rate largely agrees with previous studies of localized breast cancer, but offers the ability to track multiple variants over time, potentially providing advantages over approaches relying on only a single variant (Bettegowda et al. 2014). However, this difference is not statistically significant because of the small sample size of our study, and it would therefore be worthwhile to apply these methods to a larger cohort.

Of the four ER^+^ patients, only one had a pretreatment ctDNA fraction of >0.1%, whereas the remaining six patients had significantly higher levels (Fig. 4A,B). Increased ctDNA abundance in the TNBC and HER2^+^ tumors may be indicative of increased ctDNA release from more aggressive tumors, something seen in a recent study in lung cancer (Abbosh et al. 2017). ER^+^ patients BCR480 and BCR481 did not have detectable ctDNA at any pre-, mid-, or posttreatment time points and were therefore omitted from the serial quantification analysis. ER^+^ patient BCR488 only had detectable ctDNA in the pretreatment time point and was also omitted from the serial quantification analysis (Supplemental Table 1).

### Cell-Free DNA Concentration Increased during Treatment

**Figure 5.**
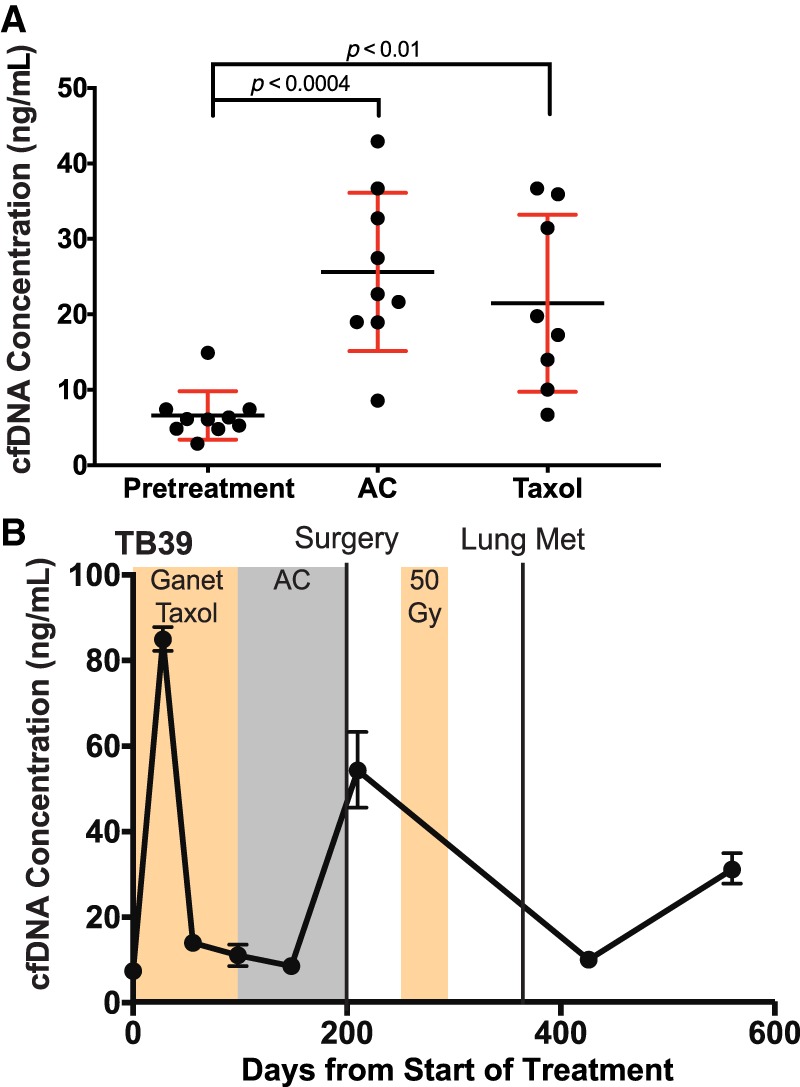
Cell-free DNA concentration increases during neoadjuvant chemotherapy. (*A*) Each point represents the average cell-free DNA concentration for all plasma samples from a single patient under a single treatment condition. Each of the ten patients had at least one pretreatment sample, and one of AC or paclitaxel treatments. Five patients have had both AC and paclitaxel treatments. Error bars ± SEM. (*B*) Serial cell-free DNA quantification of patient TB39. Second time point (day 28) corresponded with patient toxicity. (Ganet) Ganetenib, an HSP-90 inhibitor.

**Figure 6.**
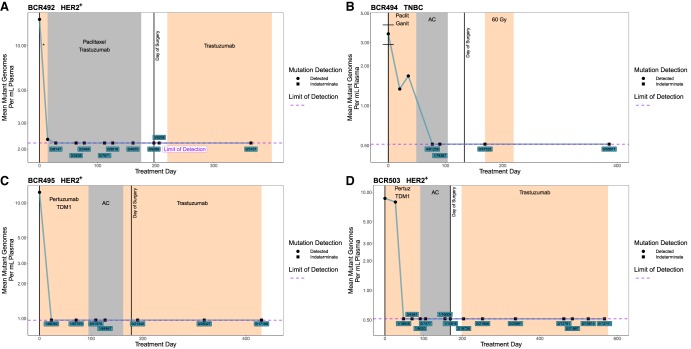
ctDNA dynamics in patients without tumor growth during NAC. Serial ctDNA quantification using patient-specific mutation panels. Limit of detection calculated as mean across all time points (see Methods). Indeterminate time points have either 0 or a statistically insignificant number of mutant reads; those points are plotted at limit of detection. Indeterminate points are labeled by mutant reads/total reads. Error bars ± SEM. (*A*) Six-mutation panel for BCR492. (*) Paclitaxel + trastuzumab + HSP90 inhibitor, which was discontinued because of toxicity issues. (*B*) Twenty-five-mutation panel for BCR494. (Ganit) Ganitumab, a monoclonal antibody targeting IGF-1R pCR, (gy) gray unit of radiation dose. (*C*) Fifteen-mutation panel for BCR495 pCR. (*D*) Eighteen-mutation panel for BCR503. (Pertuz) Pertuzumab.

Enrolled patients were treated using three different regimens based on their ER status, HER2 status, and enrollment in the ISPY2-TRIAL (Table 1) (https://clinicaltrials.gov/ct2/show/NCT01042379). For all patients, the total cell-free DNA concentration was elevated during treatment with both AC (average of fourfold) and paclitaxel (average of threefold) (Fig. 5A). Several patients saw a further spike in cell-free DNA concentration over a small number of time points and in two of those patients, BCR492 (HER2^+^) and TB39 (TNBC), had concurrent toxicity issues from treatment (Figs. 5B, 6A; Supplemental Table 1).

Variation in the amount of cell-free DNA present in the plasma before and during treatment complicates serial comparison of tumor-derived ctDNA mutant allele fractions. For example, cell-free DNA levels fluctuate significantly over multiple sampling time points as a result of healthy cell death from chemotherapy; therefore, the observed VAF of ctDNA-derived mutations does not correctly reflect true dynamics in ctDNA release from the tumor even in the case of an effective therapy. To address what are effectively uncorrelated changes in background signal, we calculated ctDNA abundance as total mutant genomes per mL of plasma, rather than simply using the VAF of a given mutation.

### ctDNA Becomes Undetectable in Patients Responding to Treatment

There were four patients who responded to NAC, indicated as pCR for patients BCR494 (TNBC) and BCR495 (HER2^+^) or decreased tumor volume for BCR492 (HER2^+^) and BCR503 (HER2^+^). In these patients ctDNA became and remained undetectable during NAC (Fig. 6). Circulating tumor DNA was undetectable for these four patients in all of the postsurgery time points and they remain disease-free for a total ctDNA-free period that ranged from ∼300 to ∼500 d from the date of last detection to the completion of this study. Patient BCR494 had ctDNA levels become undetectable during the second treatment arm (AC) (Fig. 6B), whereas the remaining three patients all had no detectable ctDNA during the first arm, which notably contained an anti-HER treatment (Fig. 6A,C,D).

### Increasing On-Treatment ctDNA Is Associated with Tumor Growth and Detectable Postsurgery ctDNA

**Figure 7.**
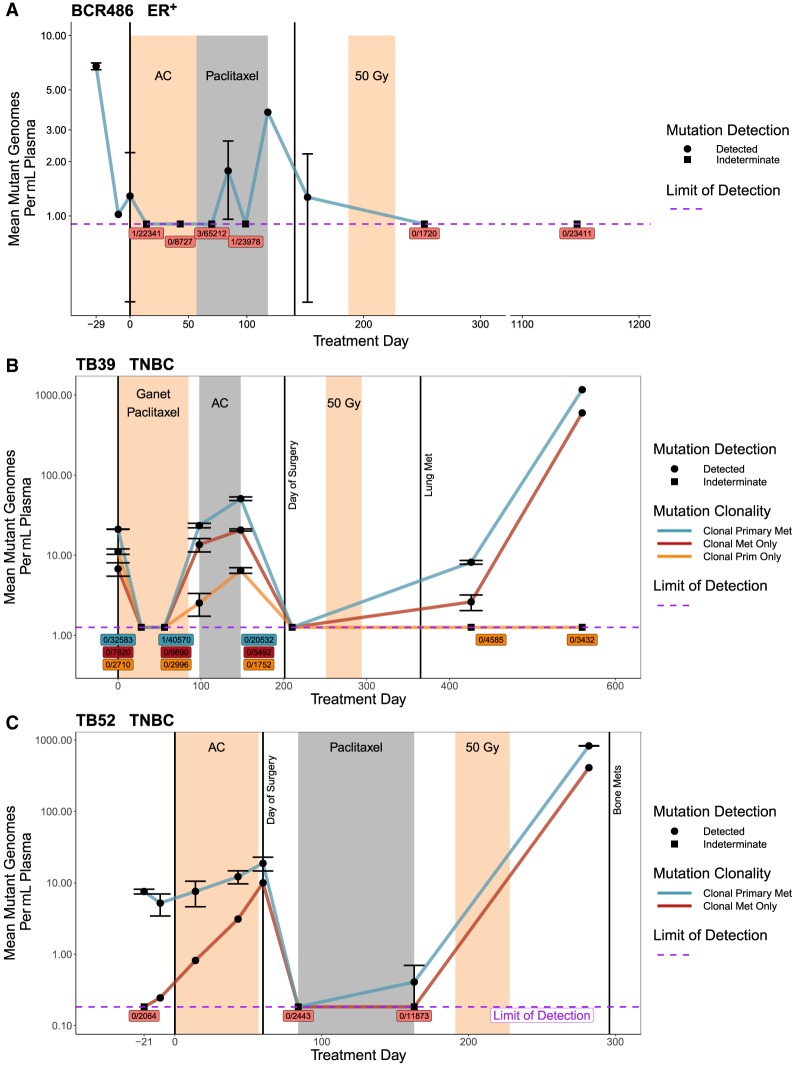
ctDNA dynamics in patients with tumor growth during NAC. Serial ctDNA quantification using patient-specific mutation panels. Limit of detection calculated as mean across all time points (see Methods). Indeterminate time points have either 0 or a statistically insignificant number of mutant reads; those points are plotted at limit of detection. Indeterminate points are labeled by mutant reads/total reads, colored by mutation clonality. Error bars ± SEM; not all time points had replicates (see Supplemental Table 2). (*A*) Nineteen-mutation panel for BCR486. Variants analyzed were observed in WES of primary tissue. (*B*) Forty-five-mutation panel for TB39. Variants analyzed were observed in WES of primary tissue and metastatic tissue. (*C*) Twenty-one-mutation panel for TB52. Variants analyzed were observed in WES of primary tissue and cell-free DNA collected concurrent with clinical detection of metastasis.

In three patients, tumor volume was larger at surgery than at the time of diagnosis (Table 1). Each of these patients showed increasing levels of ctDNA at the end of NAC and had at least one postsurgery time point with detectable ctDNA (Fig. 7). In patient BCR488, tumor size appeared to increase based on postsurgery pathology; however, pretreatment and posttreatment imaging showed a decrease in volume. This discrepancy is likely due to the variability between these two techniques (i.e., tumor volume as determined by postsurgery pathology vs. tumor volume as determined by imaging; Table 1). ctDNA become undetectable in patient BCR486 following radiation treatment and the patient remained disease-free as of the completion of this study. TNBC patients TB39 and TB52 had detectable ctDNA following adjuvant treatment, and both had a recurrence (Fig. 7B,C).

In TNBC patients TB39 and TB52 we were able use the cell-free DNA collected during occult metastatic disease and/or metastatic tumor tissue to identify a set of mutations that were clonal (having high VAF in primary and metastatic disease) as well as mutations that were present at high VAF in only the primary (clonal primary) or metastatic setting (clonal metastasis). Using these mutation classifications, we were able to track how the ctDNA abundance changed for the different populations of mutations (Fig. 7B,C). In both patients, TB39 and TB52, the clonal mutations had higher VAFs throughout treatment than those classified as clonal primary or clonal metastasis. Clonal metastasis mutations can be identified in the pre- and midtreatment setting of both patients, and in both cases, they increased in abundance during treatment. In TB39, clonal primary mutations appear less abundant than clonal metastasis mutations midway through treatment and disappear completely following surgery, suggesting a stronger response to treatment in the primary clonal compared to the metastasis clonal. Unfortunately, for TB52, we were unable to analyze primary-specific mutations because of failure of the target site capture baits. However, ctDNA levels with clonal mutations shared between the primary and metastasis as well as clonal metastasis mutations in TB52 were found to increase postsurgery, during follow-up treatment at two time points, and as early as 150 d prior to detection of bone metastasis (Fig. 7C). Unfortunately, in TB39, although ctDNA levels with clonal mutations and clonal metastasis mutations increased with the diagnosis of lung metastasis, we did not have blood draws between the postsurgery time point and that diagnosis. It is likely that we would have observed ctDNA level increase in the period prior to diagnosis of lung metastasis, based on the ctDNA levels after diagnosis (Fig. 7B).

## DISCUSSION

In this study, we demonstrate the ability to design patient-specific hybrid capture panels, using mutations called from whole-exome sequencing, to accurately detect ctDNA down to one input molecule in 10,000 before, during, and after NAC. We also observed an increase in total cell-free DNA during either paclitaxel or AC treatment, an observation that may serve as a proxy for increased death of normal cells.

A dramatic decrease in ctDNA levels occurred at the start of neoadjuvant treatment in all but one patient (TB52), eventually becoming undetectable in the four patients who did not show tumor growth during treatment. This finding is contrary to our initial hypothesis that ctDNA would become undetectable only for patients achieving pCR, and the presence or absence of midtreatment ctDNA could serve as an early indicator of pCR. Rather than serve as a marker of tumor burden, our data indicate that midtreatment ctDNA detection might instead serve as a marker of tumor growth. There are several possible mechanisms for this finding. One plausible mechanism is that chemotherapy kills the dividing cells most likely to contribute to ctDNA release and, because the half-life of cell-free DNA in the blood is very short, leaves behind a tumor less prone to ctDNA release at our sampling time of 2 wk following infusion (Lo et al. 1999). Similar results to these have been seen in the metastatic setting, in which stable disease on-treatment is accompanied by a decrease in ctDNA levels independent of changes in tumor size (Forshew et al. 2012; Dawson et al. 2013). It is possible the same situation is present in the neoadjuvant setting, but, because of the lower initial levels, ctDNA becomes undetectable in our assay. These results broadly agree with two similar studies looking at ctDNA in the neoadjuvant setting (Kim et al. 2017; Riva et al. 2017). In contrast to our study, both these studies relied on only one single on-treatment plasma sample and were therefore unable to see whether ctDNA increased over the course of NAC. Additional studies aimed specifically at understanding the mechanisms of ctDNA release and relationship between response to therapy and ctDNA levels are required to further elucidate these results.

In three patients (TB39, TB52, and BCR486), ctDNA levels increased during treatment and corresponded to increased tumor size, suggesting a role for ctDNA analysis in identifying tumor progression and assaying treatment effectiveness. Increasing ctDNA may be an indicator not only of tumor growth during treatment but of increased risk of recurrence.

Patients BCR486 and TB52 had detectable ctDNA following surgery, a result that has been shown to indicate a risk of recurrence (Garcia-Murillas et al. 2015). For TB52 this recurrence occurred just 7 mo after ctDNA detection. These results highlight a possible role for postsurgery ctDNA analysis in prediction of recurrence and assessment of adjuvant treatment effectiveness. For example, multiple adjuvant treatments might be used while monitoring ctDNA levels until they become undetectable, indicating treatment effectiveness and a reduced risk of recurrence. Results from a larger study, specifically looking at the postsurgery setting, indicated that assessing ctDNA at multiple postsurgery time points is more accurate than a single measurement and should be incorporated into future studies (Abbosh et al. 2017). It should be noted that the postsurgery follow-up time period is limited to 3 yr on average, despite the recurrence window of TNBC and HER2^+^ disease being 5 yr and for ER^+^ disease, >10 yr (Colleoni et al. 2016), making it impossible to rule out a potential recurrence in any of the other patients. It is also of note that BCR486 had a significant decrease in ctDNA levels prior to starting the AC arm of NACT. This may be a result of patient-specific disease development, atypical fluctuations in ctDNA levels, or sampling error. Again, increasing cohort size would help elucidate such dynamics.

Identifying and using variants from multiple tissue sources with this method (e.g., multiple primary lesions and/or metastasis) might reveal mechanisms for tumor evolution through clonal selection, drug resistance, and tumor growth dynamics that would be unique to individual patients. In patients TB39 and TB52 we were able to utilize metastatic tissue and/or cell-free DNA to identify a set of mutations that were clonal in the metastatic setting but were not initially observed in the primary tumor (Fig. 7B,C). The pretreatment sample for TB39 showed that mutations that were clonal in the primary but not the metastasis were initially more abundant than the clonal metastasis mutations. This pattern reverses during treatment, with the primary mutations becoming and remaining undetectable following surgery (Fig. 7B). In TB52 the clonal metastasis mutations were only observed in one of the two pretreatment ctDNA samples but increased dramatically during neoadjuvant treatment (Fig. 7C). These mutations potentially represent mutations belonging to a subclonal portion of the primary tumor, which eventually seeded the metastasis, or mutations already present in micrometastatic disease. Expanding on these results may identify patterns of mutations that are associated with increased risk of recurrence. This could also provide insight into how selection is acting upon specific mutations in various disease and treatment settings. Regional sampling of the primary tumor was not possible but could provide insight into these mechanisms and should be incorporated into future analysis.

The ER^+^ patients had significantly low ctDNA levels, potentially indicating that ER^+^ disease is particularly ill-suited for this kind of ctDNA analysis and that more sensitive assays are required (Fig. 4C). As ER^+^ tumors are generally thought to be less aggressive, ctDNA abundance may be related to tumor growth rather than simply tumor size, results which mirror those found in a previous study of lung cancer (Abbosh et al. 2017). It would also be worth exploring the relationship between initial TNM tumor stage and ctDNA, which requires a larger sample size than we had for this study.

The plasma sampling strategy we used collected blood on the day of treatment prior to chemotherapy infusion. This strategy was chosen for simplicity as the patients were already in the clinic. However, it is possible that sampling 2 wk after the previous drug administration impaired our ability to detect ctDNA. A sampling schedule that collected blood 2 or 3 d after infusion may have detected ctDNA from actively dying cells, and this signal may no longer be present 2 wk after treatment. In line with the conclusions of a larger clinical review, additional studies looking at the timing and kinetics of ctDNA release and degradation following drug administration could provide valuable insight into how to best detect on-treatment ctDNA (Merker et al. 2018). Such a study could measure the kinetics of tumor cell death and, by tracking mutations associated with subclonal populations, provide information about which cell populations were being eliminated over others.

These results highlight the potential of neoadjuvant ctDNA analysis to identify midtreatment tumor progression, measure treatment effectiveness in the adjuvant setting, and predict recurrence. Our unique DIDA error-correction sequencing method combined with tumor-specific capture panels allows for accurate detection of tumor-derived variants in cell-free DNA down to one input molecule in 10,000. Additional work is needed to expand on and replicate these findings and develop standardized methods of assaying ctDNA. It is possible that similarly promising results may be found in additional tumor types or treatment regimens, opening the door for patient-specific ctDNA analysis to contribute to individualized treatment decisions that improve clinical outcomes across other cancer types.

## Methods

### Patient Enrollment and Sample Collection

All human specimens and data were acquired from participants by obtaining informed written consent to use their coded de-identified data and/or specimens for research and publication purpose under regulation by the Oregon Health & Science University (OHSU) IRB# 8314 *Breast Cancer Registry* and the OHSU IRB# 10163 *Reconstructing the Tumor Genome in Peripheral Blood* protocols. Up to 40 mL (range 6–40 mL) of blood were collected from patients in 5 × 6-mL or 4 × 10-mL, purple-capped EDTA tubes. Consistent with the recommendations of Merker et al., within 6 h of collection blood plasma was isolated by first spinning whole blood at 1000*g* for 10 min, separating the top plasma layer into 1-mL aliquots, then spinning those aliquots at 15,000*g* for 10 min, transferring the supernatant to cryovials, and storing at −80°C (Merker et al. 2018). One patient, BCR503, had blood collected remotely, and drawn into cell-free DNA Blood Collection Tubes (Streck); samples were then shipped to us within 3 d and plasma was processed as outlined above. Buffy coat was isolated from the intermediate blood layer following the first spin and also stored at −80°C. Cell-free DNA was extracted using the QIAamp Circulating Nucleic Acid kit (QIAGEN). Buffy coat DNA was extracted using the DNA Blood Mini kit (QIAGEN).

Tumor tissue was obtained from a core needle biopsy of the primary tumor, which was placed in OCT and stored at −80°C. Prior to extraction, the OCT block was cryosectioned for pathology review. DNA was extracted using the DNeasy Blood and Tissue kit (QIAGEN). For patient TB39 we also received tumor tissue from a lung metastasis as well as metastasis tissue from TB52. For patient BCR503 we received 10 4-µm sections of an archival formalin-fixed, paraffin-embedded biopsy, and DNA was extracted using the QIAamp DNA FFPE Tissue kit (QIAGEN). DNA was quantified using the Kapa hgDNA Quantification and QC kit (Kapa Biosystems). Several high-concentration cell-free DNA samples were subsequently tested on the Bioanalyzer 2100 to check for the presence of high-molecular-weight DNA, likely from lysed blood cells.

### Whole-Genome and Whole-Exome Sequencing and Analysis

Genomic DNA extracted from BCR488 was sent to the Broad Institute for WGS, and data was delivered via secure ftp. Whole-exome sequencing (WES) was conducted on matched buffy coat and primary tumor DNA for all patients. For patients TB39 and TB52 whole-exome sequencing was also conducted of cfDNA collected during occult metastatic disease. Finally, WES was conducted on metastatic tumor DNA for TB39 as well. All WES was carried out using HPLC-purified, dual-index adapters ordered from IDT (idtdna.com). These were used in combination with the Hyper Prep DNA Library Preparation Kit (Kapa Biosystems), at a 10:1 adapter:template ratio. This library was then used as input for the Agilent SureSelect XT hybrid-capture protocol and reagents using the human all-exon v5 set of capture baits (Agilent). These libraries were either sequenced using the Illumina HiSeq 2500 platform (paired-end 100 bp), or the Illumina NextSeq 500 platform (paired-end, 75 bp). FastQ data files for all WES and WGS were aligned using the reference genome, hg19 (Genome Reference Consortium Human Build 37 [GRCh37]) with BWA MEM (0.7.12, GATK, Broad Institute; read counts, alignment, and coverage analysis are found in Supplemental Table S3). Somatic mutations were called using MuTect (1.1.9, GATK, Broad Institute) using the associated buffy coat sample as a matched normal (Cibulskis et al. 2013). Mutations were filtered such that each had a VAF of 0.1 and at least three supporting reads with bidirectional support (Supplemental Table S2). Twenty to 50 mutations for each patient were chosen to be included in the patient-specific DIDA panels. Recurrent mutations in breast cancer divers (*PIK3CA, TP53, ATK1*) were always included when present. Additional passenger mutations were chosen to prefer mutations present at high VAF, and, when possible, to avoid T > C or G > A mutations, which consistently have higher error rates (Supplemental Table S1).

### DIDA Library Preparation and Sequencing

DIDA error-correction libraries were created using the Kapa Biosystems Hyper Prep kit using at least 30 ng of cell-free DNA as input (Fig. 2). DIDA oligonucleotides containing 4- to 6-bp degenerate sequences as unique molecular identifiers (UMIs) were purchased from IDT (idtdna.com). Ligation occurred using a 200:1, adapter:template ratio for 16 h at 16°C ensure a high-efficiency ligation. PCR was conducted to generate a 1-μg library (typically eight to 10 cycles). Library concentration and size was determined using the Agilent Bioanlyzer 2100 high-sensitivity kit. Then 250 ng of the library was combined with 250 ng from a different sample and input into the duplexed hybrid capture, allowing the remaining 750 ng to be used for subsequent hybrid captures. Hybrid capture was conducted using the IDT Hybridization and Wash kit.

Following the first 4-h hybridization and capture, libraries were amplified for 11 cycles and purified using Agencourt AMPure XP beads (Beckman Coulter, Inc.). The library was hybridized, captured a second time, and amplified for an additional 11 cycles. Library size was determined using the Agilent Bioanalyzer 2100 high-sensitivity kit and concentration was determined using the Kapa Biosystems Library Quantification Kit. Samples were sequenced on either the Illumina HiSeq 2500, paired-end 100 bp, with dual 14-bp indexing cycles (high-capacity, rapid run mode) or the Illumina NextSeq 500, paired-end 75 bp with dual 14-bp indexing cycles (high-capacity, 150-cycle kit).

### Safe-SeqS Library Preparation and Sequencing

The Safe-SeqS library was prepared as described previously (Kinde et al. 2011). Briefly, a pair of mutation-specific primers were ordered that, in addition to 20 template-specific bases, contained 12 degenerate Ns and the first half of the Illumina Sequencing adapter. This was subjected to four cycles of PCR using Phusion Hotstart II polymerase. Following PCR, unused primers were removed using RecJf nuclease, and cleaned up using AMPureXP beads. A second round of 30 PCR cycles was conducted using primers against the Illumina sequence and containing an Illumina sample index and the remainder of the adapter. This was then purified again with AMPureXP beads, sequenced spiked-in to another library at <1%, and sequenced on the Illumina NextSeq 500. This method was used only on patient BCR 486 for assaying a *PIK3CA* H1047R mutation (Supplemental Table S2).

### DIDA Sequencing Analysis

The pipeline for analyzing DIDA data was based on the duplex sequencing pipeline developed in the Loeb laboratory at the University of Washington with substantial modification to be compatible with our data (Cibulskis et al. 2013). In brief, indexing reads (containing sample index and degenerate barcode) were appended to the read header of each of the paired-end reads. MIGEC Checkout was used to demultiplex the samples using the fixed indexing barcode (Shugay et al. 2014). Next, a modified version of the duplex sequencing pipeline was used to align the paired-end reads and generate and realign consensus sequences (Fig. 2). The pipeline aligned the reads using BWA MEM, grouped the reads by mapping position and degenerate barcode, and then collapsed them into a SSCS requiring at least three reads and 90% agreement between reads, otherwise resulting in read omission or an “N” at a given consensus site, respectively. SSCSs were then realigned using BWA MEM, five bases from either end were replaced with Ns to remove low-quality bases, and overlapping reads were collapsed to avoid double-counting (bamUtil clipOverlap) (Breese and Liu 2013). Finally, consensus sequences thought to derive from same initial molecule, but missed because of index hopping or errors in the degenerate barcode, were filtered out using a Python Script. Briefly, this script (1) identifies SSCSs that have the exact same start and stop position, and (2) removes SSCSs that have an exact match in one of the two 6-bp degenerate barcodes. This will remove SSCSs differing by a single base (which was likely introduced via a PCR or sequencing error) and SSCSs that underwent a “tag swap” (as a result of via PCR-mediated recombination). In either case, SSCSs that are misidentified as coming from independent molecules are removed.

### Data Availability

All sequencing alignment bam files generated by whole-exome and whole-genome sequencing, as well as by DIDA-custom target panel testing and time points for individual patients, are available in the Sequence Read Archive (www.ncbi.nlm.nih.gov/sra) under the eSRA project accession # PRJNA516884 or at the following link: https://www.ncbi.nlm.nih.gov/sra/?term=PRJNA516884 (Supplemental Table S4).

### Panel Evaluation

For each patient-specific panel, SSCSs from all negative control experiments were pooled (averaging 200,000× depth). High error sites (those with VAFs > 0.05 in the negative control samples) were predominantly the result of mismapping and had been seen in multiple unrelated sequencing experiments. These mutations were removed from the subsequent analysis. Error rates varied in different sequence contexts with C > G having the highest error rate and A > G having the lowest error rate (Supplemental Fig. 1). For each patient-specific panel, the panel-wide error rate was determined by summing the total WT depth across all negative control runs and all sites and then dividing by the number of observed variants matching the base of interest present in the patient's tumor. Using this panel-wide error rate, a 95% confidence interval was generated, and the upper bound was used as a conservative estimate of the panel's true error rate (Supplemental Table S1).

### Mutant Genome Calculation

For each time point and replicate, ctDNA allele frequencies were determined by summing the total mutant reads across the patient-specific panel and dividing by the total depth across the panel. The number of observed mutant reads was compared to the number expected by chance using a binomial test and to the panel's error rate. Only time points with *P* < 0.05 were considered true positives (Supplemental Table S2). Because of variation in the amount of cell-free DNA assayed, number of variants in the panel, and performance of the library construction, the actual limit of detection for each time point differed. We have indicated when a time point is below the technical limit of detection (sufficient sequencing depth but 0 or a statistically insignificant number of observed mutant reads) versus indeterminate (0 or a statistically insignificant number of observed mutant reads, but a sequencing depth above our technical limit of detection). To correct for differences in overall cell-free DNA concentration, the VAF was converted into mutant genomes by the following equation:

$$\begin{array}{rl} & \frac{\mathrm{cfDNA}\;\mathrm{concentration}\left(\frac{\mathrm{ng}}{\mathrm{mL}\;\mathrm{plasma}}\right)}{0.003\left(\frac{\mathrm{ng}}{\mathrm{genome}}\right)}*\mathrm{variant}\;\mathrm{allele}\;\mathrm{frequency}\\ & =\mathrm{Mutant}\;\mathrm{genomes}\;\mathrm{per}\;\mathrm{mL}\;\mathrm{plasma}. \end{array}$$

### Limit of Detection Calculation

For each time point, a limit of detection was calculated based on the error rate of the panel and sequencing depth at that time point. In short, we determined the minimal number of reads required for a binomial test to be significant given the panel error rate and sequencing depth achieved. This was then converted into a VAF based on the sequencing depth and converted to mutant genomes using the formula above. The per-time point limit of detection values are in Supplemental Table S2. For ease of plotting, the per-time point values were then averaged to a mean limit of detection for display in Figures 6 and 7.

## ADDITIONAL INFORMATION

### Data Deposition and Access

We have made the de-identified, aligned sequencing data for all relevant sequencing done for the experiments and analysis presented in this manuscript available at the eSRA website (https://www.ncbi.nlm.nih.gov/sra/) under project accession number PRJNA516884.

### Ethics Statement

This study was approved by the Institutional Review Boards of the Oregon Health Science University. All human specimens and data were acquired from participants by obtaining informed consent for genetic testing under regulation by the Oregon Health & Science University (OHSU) IRB# 8314 Breast Cancer Registry & OHSU IRB# 10163 Reconstructing the Tumor Genome in Peripheral Blood protocols. All participants consented to the use of their coded de-identified data and/or specimens for publication purposes. Research was conducted in accordance with the International Conference on Harmonization (IHC) E6 GCP guidelines and the FDA. Written informed consent was obtained from all patients for publication of their de-identified individual and clinical details and associated genetic data in this manuscript as described in the “Ethics approval and consent to participate” section above. The consent form is held by the authors and by the OHSU IRB and is available for review by the Editor-in-Chief.

### Author Contributions

T.M.B. was responsible for experimental design and development of the DIDA-error correction sequencing methods, prepared and sequenced patient DNA, and analyzed sequencing data. C.T.B. carried out evaluation of competing error-correction methods and follow-up data analysis, pipeline testing and development assistance, and data wrangling. K.J.-C. authored and managed relevant IRB protocols and sample collection, which included patient identification, informed consent, patient tracking, blood and tissue collection, and blood and tissue processing. S.T. assisted with the patient tracking, blood and tissue collection, and blood and tissue processing. T.K. and D.M. assisted with long-term follow-up patient tracking, blood collection, and blood processing. J.G. oversaw protocol development. C.L.C. carried out pathological review of the tumor specimen and assisted with tumor sample acquisition, FFPE preservation, and sectioning. P.T.S. oversaw this project in its entirety. T.M.B. and C.T.B. composed this manuscript and designed and developed its accompanying figures and tables. All authors read and approved the final manuscript.

### Funding

This work was supported by the Circle of Giving and the OHSU Center for Women's Health, the Prospect Creek Foundation, and the OHSU Department of Medical and Molecular Genetics Research Startup Funding awarded to Dr. Paul Spellman. These funders had no role in the preparation, review, or approval of the manuscript nor in the decision to submit the manuscript for publication.

### Competing Interest Statement

The authors have declared no competing interest.

## Supplementary Material

Supplemental Material
