# Supplementary material for: Circulating tumor DNA dynamics using patient-customized assays are associated with outcome in neoadjuvantly treated breast cancer
Source: Cold Spring Harb Mol Case Stud. 2019 Apr;5(2):a003772. doi: 10.1101/mcs.a003772 (PMC6549569; doi:10.1101/mcs.a003772)
Supplement: Supplemental Material [file supp_5_2_a003772__index.html]

Circulating tumor DNA dynamics using patient-customized assays are associated with outcome in neoadjuvantly treated breast cancer — Supplemental Material 

# Circulating tumor DNA dynamics using patient-customized assays are associated with outcome in neoadjuvantly treated breast cancer

## Supplemental Material

- Supplemental\_FigS1.pdf
- Supplemental\_Legends.docx
- Supplemental\_Table\_S1.xlsx
- Supplemental\_Table\_S2.xlsx
- Supplemental\_Table\_S3.xlsx
- Supplemental\_Table\_S4.xlsx
