# Supplementary figures and images for: Circulating tumor DNA dynamics using patient-customized assays are associated with outcome in neoadjuvantly treated breast cancer
Source: Cold Spring Harb Mol Case Stud. 2019 Apr;5(2):a003772. doi: 10.1101/mcs.a003772 (PMC6549569; doi:10.1101/mcs.a003772)

A.

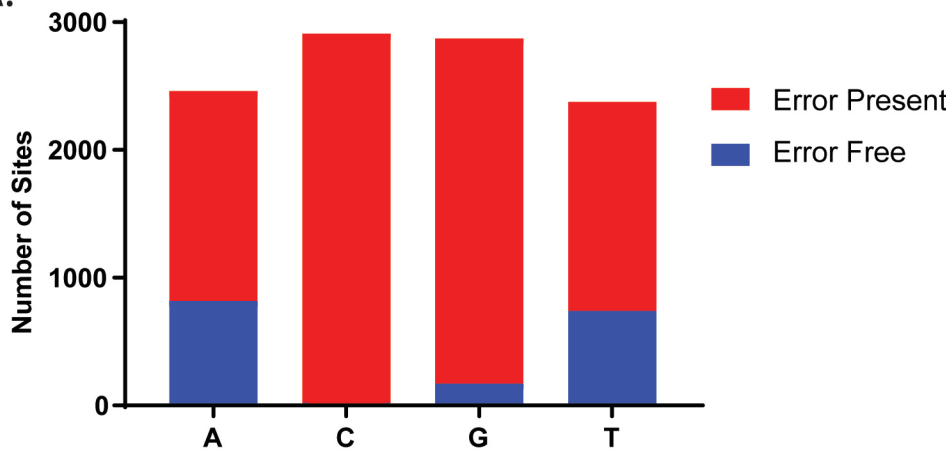

B.

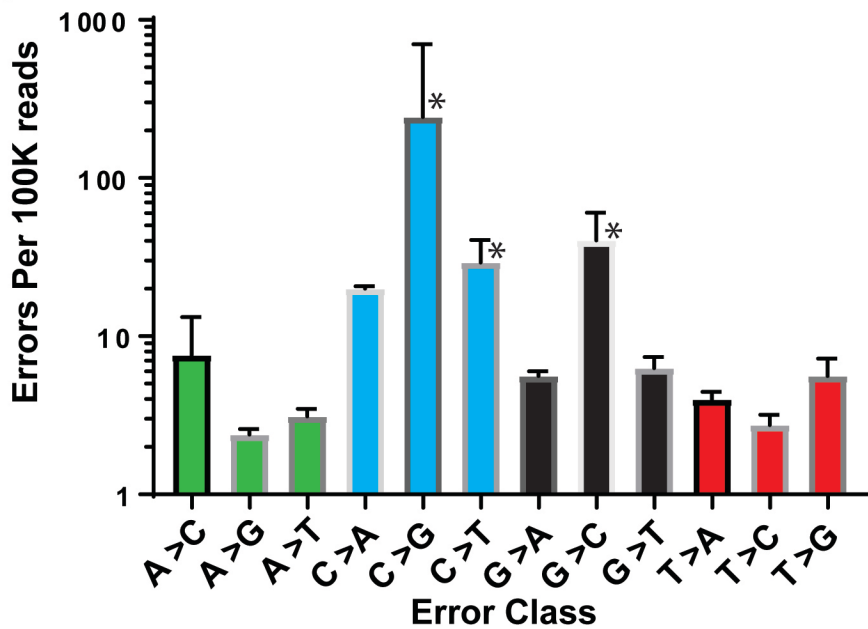

Supplement: Supplemental Material [file supp_mcs.a003772_Supplemental_FigS1.pdf]
