# Supplementary material for: Circulating tumor DNA dynamics using patient-customized assays are associated with outcome in neoadjuvantly treated breast cancer
Source: Cold Spring Harb Mol Case Stud. 2019 Apr;5(2):a003772. doi: 10.1101/mcs.a003772 (PMC6549569; doi:10.1101/mcs.a003772)
Supplement: Supplemental Material [file supp_mcs.a003772_Supplemental_Legends.docx]

**Supplemental Files**

**Supplemental Figure S1** Distribution of sequencing errors. A) Comparison of the number of sites which are error-free based on the identity of the WT base. All pairwise comparisons except A-T are statistically significant (p<0.0001). B) Median error frequency for each class of sequencing error. Asterisks indicate that class is significantly different from all none asterisk classes. Error bars 95% CI.

**Supplemental Table S1** Patient-Specific Panel Error Rates. Summary of mutations included in each patient-specific panel, and error rates in negative control experiments.

**Supplemental Table S2** ctDNA Measurements. Summary of serial ctDNA measurement for each patient.

**Supplemental Table S3** Whole exome and whole genome sequence statistics

**Supplemental** **Table S4** NCBI eSRA file accession numbers
